# Autosomal recessive OSMRβ deficiency: Connecting OSM and/or IL-31 with atopy

**DOI:** 10.70962/jhi.20260115

**Published:** 2026-08-19

**Authors:** Anne Puel, Jean-Laurent Casanova, Vivien Béziat

**Affiliations:** 1 Université Paris Cité, Institut Imagine, Laboratory of human genetics of infectious diseases, Necker branch, INSERM UMR 1163, F-75015, Paris, France; 2 Pediatric Hematology-Immunology and Rheumatology Unit, Necker Hospital for Sick Children, Assistance Publique-Hôpitaux de Paris, Paris, France; 3 Laboratory of Human Genetics of Infectious Diseases, UT Southwestern Branch, Dallas, TX, USA; 4 Howard Hughes Medical Institute, New York, NY, USA; 5 Center for Genetics of Host Defense, UT Southwestern, Dallas, TX, USA; 6 Departments of Pediatrics and Immunology, UT Southwestern, Dallas, TX, USA; 7 Children Research Institute, UT Southwestern, Dallas, TX, USA

## Abstract

Hyper-IgE syndrome (HIES) is characterized by recurrent infections, severe eczema, impaired inflammation, and extrahematopoietic manifestations. Most patients carry dominant-negative *STAT3* variants, which impair IL-6 family cytokine signaling. In this News & Views, we discuss two studies reporting autosomal recessive (AR) OSMRβ deficiency as a new inborn error of immunity. All 11 patients had severe atopy, hyper-IgE, and eosinophilia; one also had HIES-like infections and extrahematopoietic features. OSMRβ (encoded by *OSMR*) and gp130 form the OSM receptor II (LIFR and gp130 form OSMR I), while OSMRβ and IL-31RA form the IL-31 receptor. Patients’ *OSMR* variants impair OSM-induced STAT activation; IL-31 signaling was not tested. It was reported that AR OSM deficiency causes bone marrow failure, while an IL-31RA–blocking antibody improves atopic dermatitis. The respective contributions of altered OSM and IL-31 signaling to atopy in AR OSMRβ deficiency remain unresolved. These findings expand the genetic dissection of the STAT3-HIES spectrum. AR OSMRβ deficiency should be considered in patients with one or more HIES-like features.

## Teaser

In this News & Views, we discuss two studies reporting autosomal recessive OSMRβ deficiency as a new inborn error of immunity marked by severe atopy, hyper-IgE, and eosinophilia, thereby refining the genetic dissection of STAT3-dependent hyper-IgE syndrome.

Hyper-IgE syndrome (HIES) is defined by a constellation of features including severe eczema, recurrent bacterial and fungal infections of the skin and lungs, poor clinical and biological inflammatory responses, high serum IgE concentrations, eosinophilia, and extrahematopoietic manifestations, including connective tissue abnormalities affecting skeletal, dental, and vascular structures (1, 2, 3). The seminal identification of germline dominant-negative variants of *STAT3* as genetic etiologies of HIES suggested that impaired signaling by STAT3-activating cytokines was a central mechanism of the disease (4). These cytokines include members of the IL-6 and IL-10 families and, to a lesser extent, those of the IL-12 family, γc-dependent cytokines, type I and III interferons, and colony-stimulating factors (5). Subsequent genetic and mechanistic studies identified additional etiologies, including autosomal recessive (AR) and dominant partial deficiencies of IL-6ST/gp130, the shared signal-transducing subunit of the IL-6 cytokine family, and AR deficiency of ZNF341, a transcription factor required for normal STAT3 transcription and activity (6, 7, 8, 9, 10, 11). Together, these disorders confirm the key role of defective STAT3-dependent signaling in HIES while implicating IL-6 family cytokines as key upstream drivers of disease. Additional inborn errors of immunity (IEIs) affecting individual IL-6 family cytokines or their receptors have provided a unique opportunity to dissect the physiological roles of specific cytokine pathways in humans, revealing a spectrum of disorders with different or partially overlapping phenotypes (12, 13, 14) (Table 1).

**Table 1.** IEIs and HIES-like phenotypes

Genetic defect	*STAT3*	*ZNF341*	*IL6ST*		*IL6R*	*OSMR*
Inheritance	AD	AR	AR partial	AD	AR	AR
Core syndrome	HIES	HIES	HIES	HIES	HIES-like	Severe atopy/HIES-like
Skin and pulmonary infections	+	+	+	+	+	+/−
Atopic dermatitis/eczema	+	+	+	+	+	+
Extrahematopoietic/developmental abnormalities	+	+	+	+	−	+/−
Hyper-IgE	+	+	+	+	+	+
Eosinophilia	+	+	+	+	+	+
Low levels of memory B cells	+	+	+	+	+	+/−
Low levels of Th17 cells	+	+	+/−	+/−	+/−	−
Impaired acute-phase/inflammatory responses	+	+/−	+	+/−	+	ND/not reported
Impaired IL-6 signaling	+	+	+	+	+	−
Impaired IL-11 signaling	ND/expected	ND/expected	+	+	ND/not expected	−
Impaired LIF signaling	ND/expected	ND/expected	+/−	+/−	ND/not expected	−
Impaired OSM signaling	ND/expected	ND/expected	+/−	+/−	ND/not expected	+
Predominant compartment	Hematopoietic/stromal/epithelial	Hematopoietic/stromal/epithelial	Hematopoietic/stromal/epithelial	Hematopoietic/stromal/epithelial	Hematopoietic/stromal/epithelial	Stromal/epithelial
Key references	(4)	(9, 10)	(6, 7, 8)	(11)	(15, 16)	(17, 18)

AD, autosomal dominant; AR, autosomal recessive; HIES, hyper IgE syndrome; IEI, inborn error of immunity; ND, not determined.

**Figure 1.**
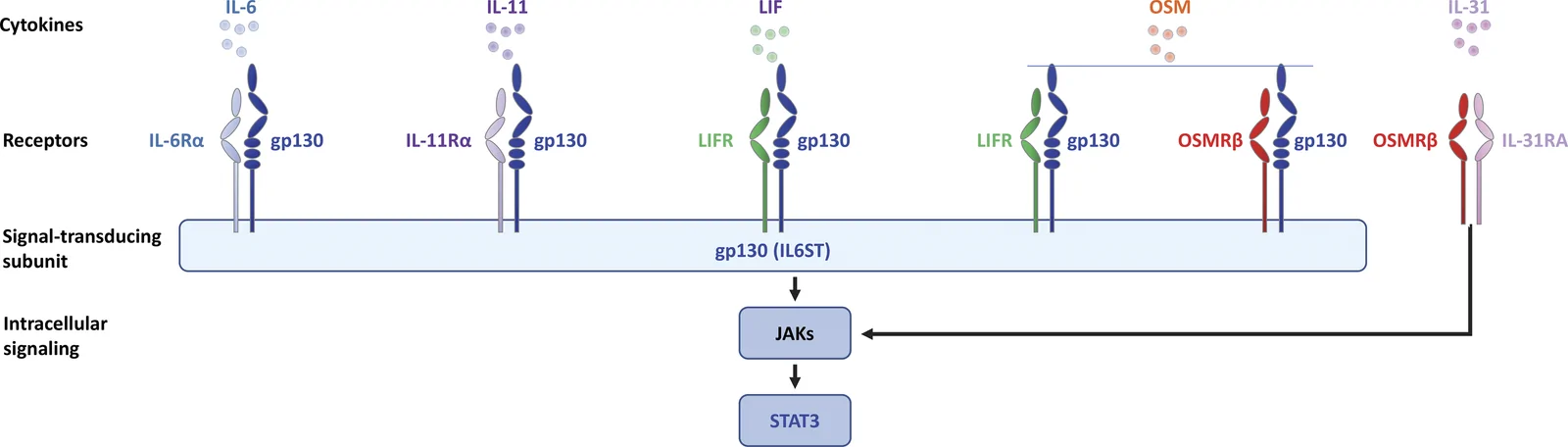
IL-6 family cytokine pathways involved in the genetic dissection of the STAT3-HIES phenotype.

In humans, IL-6, IL-11, leukemia inhibitory factor (LIF), oncostatin M (OSM), CT-1, CLCF1, CNTF, and IL-27 stimulate receptor complexes containing IL-6ST/gp130 (19). AR IL-6R deficiency linked impaired IL-6 signaling to recurrent bacterial infections, defective acute inflammatory responses, high IgE levels, eosinophilia, and atopic disease (15, 16), whereas AR IL-11RA deficiency associated impaired IL-11 signaling with craniosynostosis, delayed tooth eruption, dental abnormalities, and variable skeletal/connective tissue manifestations (20, 21). AR LIF receptor (LIFR) deficiency underlies Stüve–Wiedemann syndrome, a severe skeletal dysplasia characterized by neonatal respiratory distress, dysautonomia, feeding difficulties, and early mortality, thereby highlighting the essential developmental role of LIFR-dependent cytokine signaling (22). A closely overlapping, even more severe, lethal Stüve–Wiedemann-like phenotype is observed in patients with complete IL-6ST/gp130 deficiency, consistent with the shared requirement for IL-6ST/gp130-dependent signaling downstream from LIFR-containing receptor complexes, together with the broader loss of gp130-dependent cytokine responses (23). AR IL-27RA deficiency revealed the nonredundant role of IL-27 in protective immunity to Epstein-Barr virus (EBV) infection (24). AR OSM deficiency causes a severe inherited bone marrow failure syndrome, with profound anemia, thrombocytopenia, and neutropenia, probably due to a disruption of the OSM-dependent support of hematopoiesis within the bone marrow environment (25). Collectively, these IEIs suggest that many hallmark features of STAT3-deficient HIES are due to the combined disruption of multiple IL-6 family cytokine pathways (Tables 1 and 2). In previous issues of *JHI*, two independent studies by Andersen and coworkers (17) and Samra and coworkers (18) identified biallelic loss-of-function variants of *OSMR* in patients with severe atopy, a phenotype overlapping that seen in patients with dominant-negative *STAT3* variants; one patient also displayed severe infections and extrahematopoietic manifestations resembling classical HIES. These findings add a new layer to the genetic and mechanistic dissection of STAT3-dependent conditions, and shed light on the contribution of OSM receptor β (OSMRβ)–dependent signaling to human immunity and allergic inflammation within the broader IL-6 cytokine family (Fig. 1 and Table 3).

**Table 2.** Additional inborn errors of IL-6 family cytokines/receptors

Genetic defect	*IL6ST*	*IL11RA*	*LIFR*	*OSM*	*OSMR* a	*IL31RA* a	*IL27RA*
Inheritance	AR complete	AR	AR	AR	AD	AD	AR
Core syndrome	SWS	Craniosynostosis/dental abnormalities	SWS	Severe bone marrow failure	FPLCA	FPLCA	EBV susceptibility
Main clinical lesson	IL-6ST/gp130 is essential for skeletal/autonomic development	IL-11 controls craniofacial/dental development	LIFR is essential for skeletal/autonomic development	OSM supports hematopoiesis/bone marrow niche	OSMRβ skin axis implicated in pruritic amyloidosis	IL-31RA skin axis implicated in pruritic amyloidosis	IL-27 is nonredundant in anti-EBV immunity
Skin and pulmonary infections	Not reported	Not reported	Not reported	Not reported	Not reported	Not reported	Not prominent
Atopic dermatitis/eczema	+/−	Not reported	Not reported	Not reported	± association reported	Not reported	Not reported
Extrahematopoietic/developmental abnormalities	Skeletal dysplasia/neonatal lung dysfunction	Craniosynostosis, dental abnormalities	Skeletal dysplasia, pulmonary dysfunction, dysautonomia, urinary tract malformation	Not reported	Skin-limited amyloidosis; eosinophilic material in the skin	Skin-limited amyloidosis	Not reported
Hyper-IgE	Not reported	Not reported	Not reported	Not reported	Not reported	Not reported	Not reported
Eosinophilia	Not reported	Not reported	Not reported	Not reported	Not reported	Not reported	Not reported
Low memory B cells	Not reported	Not reported	Not reported	Not reported	Not reported	Not reported	+
Low Th17 cells	Not reported	Not reported	Not reported	Not reported	Not reported	Not reported	−
Impaired acute-phase/inflammatory responses	+	Not reported	Not reported	Not reported	Not reported	Not reported	Not reported
Impaired IL-6 signaling	+	−	ND	ND	ND	ND	−
Impaired IL-11 signaling	+	+	ND	ND	ND	ND	ND
Impaired LIF signaling	+	−	+	ND	ND	ND	ND
Impaired OSM signaling	+	−	ND	+ (ligand absent)	+/−	ND/not expected	ND
Impaired IL-31 signaling	ND	ND	ND	ND	+/−	ND/possibly affected in skin	ND
Impaired IL-27 signaling	+	−	ND	ND	ND	ND	+
Predominant compartment	Skeletal/mesenchymal	Craniofacial/mesenchymal	Skeletal/autonomic/mesenchymal	Hematopoietic niche	Skin/epithelial-stromal	Skin/epithelial-stromal	T cell anti-EBV immunity
Key references	(23)	(6, 20, 21)	(22)	(25)	(26, 27, 28)	(27)	(24)

AD, autosomal dominant; AR, autosomal recessive;  SWS, Stüve–Wiedemann syndrome; FPLCA, familial primary localized cutaneous amyloidosis; EBV, Epstein-Barr virus; ND, not determined.

a *OSMR* and *IL31RA* variants have been reported in the heterozygous state in patients with FPLCA, their functional consequences appear context- and variant-dependent and remain incompletely defined.

**Table 3.** Genetic dissection of the STAT3-HIES phenotypes based on inborn errors of the IL-6 family cytokine pathway

Deficiency	AR complete IL-6ST deficiency	AR partial IL-6ST deficiency	AD partial IL-6ST deficiency	AR IL-6Rα deficiency	AR IL-11Rα deficiency	AR LIFR deficiency	AR OSM deficiency	AR OSMRβ deficiency	*OSMR* missense heterozygous variants (FPLCA)	*IL31RA* missense heterozygous variants (FPLCA)
Affected signaling axis	Complete loss of multiple gp130-dependent cytokines	Partial defect of multiple gp130-dependent cytokines	Partial defect of multiple/specific gp130-dependent cytokines	IL-6 signaling	IL-11 signaling	LIFR-dependent cytokine signaling	OSM production/OSM signaling	OSM signaling; IL-31 R signaling likely altered	*OSMR*/IL-31 skin axis	*IL31RA*/IL-31 skin axis
Major clinical manifestations	Severe/frequently lethal SWS-like syndrome, neonatal respiratory dysfunction, skeletal abnormalities	HIES-like disease with recurrent infections, atopic dermatitis, hyper-IgE, eosinophilia, impaired acute-phase responses, craniosynostosis/developmental features	HIES-like disease with recurrent sinopulmonary infections, severe pulmonary complications, hyper-IgE/eosinophilia, retained deciduous teeth, skeletal/connective tissue features	Recurrent bacterial infections, defective acute inflammation, atopy, hyper-IgE, eosinophilia	Craniosynostosis, delayed tooth eruption, dental abnormalities, skeletal/connective tissue features	SWS, skeletal dysplasia, respiratory distress, dysautonomia, feeding difficulties, early death	Severe bone marrow failure, anemia, neutropenia, thrombocytopenia	Severe atopic dermatitis, hyper-IgE, eosinophilia, ± infections and HIES-like features	Pruritus, primary localized cutaneous amyloidosis, ± atopic dermatitis	Pruritus, primary localized cutaneous amyloidosis, ± atopic dermatitis

AD, autosomal dominant; AR, autosomal recessive; FPLCA, familial primary localized cutaneous amyloidosis; SWS, Stüve–Wiedemann syndrome; HIES, hyper-IgE syndrome.

OSM is an IL-6 family cytokine produced principally by activated hematopoietic cells, including T cells, monocytes/macrophages, neutrophils, and dendritic cells (29, 30, 31). It signals through receptor complexes containing IL-6ST/gp130. It can engage two receptor complexes: the type II OSM receptor, composed of OSMRβ and IL-6ST/gp130, and the type I OSM receptor, composed of LIFR and IL-6ST/gp130 (32, 33). The engagement of these receptors activates JAK-dependent pathways, particularly the STAT3 pathway, but also the STAT1, STAT5, MAPK, and PI3K signaling pathways (32, 33, 34). OSM is a pleiotropic cytokine. Indeed, OSMRβ is expressed in many different nonhematopoietic cell types, including fibroblasts, keratinocytes, epithelial cells, endothelial cells, osteoblasts, and other mesenchymal cells (35). LIFR is also expressed in a broad range of nonhematopoietic cells, overlapping substantially with OSMRβ-expressing stromal, epithelial, endothelial, and mesenchymal cell types (36). OSM has been implicated in wound repair, extracellular matrix remodeling, fibrosis, bone metabolism, endothelial activation, and inflammatory responses (34, 37). In fibroblasts and other stromal or epithelial cells, OSM induces transcriptional programs involving cytokines, chemokines, interferon-responsive genes, matrix remodeling factors, and barrier-associated pathways (34). OSMRβ also forms the heterodimeric IL-31 receptor together with IL-31RA (34, 38). IL-31 is produced predominantly by activated Th2 lymphocytes and signals via JAK-STAT pathways, with important roles in pruritus, epithelial inflammation, and atopic skin disease (39, 40). OSMRβ therefore lies at the intersection of two cytokine pathways, the OSM and IL-31 pathways, linking cytokine-driven STAT3-dependent responses to epithelial, stromal, and tissue remodeling responses.

In total, Andersen, Samra, and their coworkers studied 11 individuals from eight unrelated families of European, South Asian, or Arab ancestry (17, 18). All patients carried biallelic in-frame or out-of-frame *OSMR* variants affecting the extracellular domain of OSMRβ. These variants clustered within the cytokine-binding or fibronectin type III–like (FNIII) domains, which are essential for receptor assembly and downstream signaling. Seven patients carried biallelic predicted loss-of-function (pLOF) variants, whereas four patients carried the p.Val436Asp allele, three in the homozygous state and one as a compound heterozygote. This variant is relatively frequent in population databases (minor allele frequency [MAF] of 3.39 × 10^−3^ in gnomAD v4.1.0) and has a high Combined Annotation Dependent Depletion (CADD) score of 24.3; gnomAD includes 14 homozygous individuals, including nine from the UK Biobank. Available UK Biobank data indicate that three of these nine homozygotes have features suggestive of allergic disease, including high eosinophil counts, allergic manifestations, or skin phenotypes. These observations support the clinical relevance of p.Val436Asp, while also suggesting variable expressivity and possibly incomplete penetrance. Moreover, 10 other missense variants with CADD scores above the mutation significance cutoff of 19.3, six of which are located in FNIII domains, have also been reported in the homozygous state in gnomAD, with MAF ranging from 2.54 × 10^−2^ to 3.72 × 10^−6^. It will be important to delineate the phenotype of homozygotes or compound heterozygotes.

A key mechanistic insight from both studies is that patient-derived *OSMR* variants selectively impair OSM-induced STAT1, STAT3, and STAT5 phosphorylation, while preserving signaling downstream from other gp130-dependent cytokine receptors, including IL-6Rα, IL-11Rα, and LIFR, together with IL-27Rα/gp130-mediated STAT1 activation. OSM-induced signaling was markedly reduced but not abolished, consistent with the ability of OSM to signal also through LIFR–gp130 complexes, and with the absence, in OSMR-deficient patients, of the severe bone marrow failure observed in patients with inherited OSM deficiency (25). In both overexpression systems and patient-derived fibroblasts, the patients’ variants markedly reduced or abolished OSMRβ surface expression, greatly decreasing OSM-induced STAT1, STAT3, and STAT5 activation, whereas responses to IL-6, IL-11, IL-27, and LIF remained largely intact. The tested variants found in the homozygous state in gnomAD, with the notable exception of p.Val436Asp, retained normal OSMRβ surface expression and signaling in overexpression systems. A causal link between the genotype and phenotype was further supported by the restoration of OSM signaling upon re-expression of the wild-type receptor, whereas fibroblasts from heterozygous carriers were similar to those of controls, consistent with recessive inheritance. OSMRβ is also involved in IL-31 receptor signaling, but the impact of the patients’ variants on IL-31 responses was not tested. Nevertheless, altered IL-31 receptor biology may contribute to some of the atopic and epithelial features observed.

Indeed, the distinctive clinical manifestations associated with AR OSM deficiency, AR OSMRβ deficiency, and heterozygous *OSMR* variants associated with familial primary localized cutaneous amyloidosis (FPLCA), a chronic pruritic skin disorder characterized by the localized dermal deposition of keratinocyte-derived amyloid (26, 27), suggest that impaired OSM signaling alone may not fully account for the severe atopic manifestations observed in patients with AR OSMRβ deficiency. Several observations support this interpretation. First, AR OSM deficiency causes severe bone marrow failure but has not been associated with atopy, whereas AR OSMRβ deficiency causes severe atopy without the bone marrow failure observed in OSM-deficient patients (25, 27, 28). Second, a phase II trial of an OSM-blocking monoclonal antibody reported hematologic, but not atopic, adverse events (41). Mechanistically, OSM signaling is likely to be partially preserved in individuals with AR OSMRβ deficiency through the type I OSM receptor, composed of LIFR and gp130. In contrast, IL-31 signals through a single known receptor complex composed of IL-31RA and OSMRβ, and compensatory signaling is therefore less likely (38). This raises the possibility that altered IL-31R signaling contributes to the cutaneous phenotype of patients with AR OSMRβ deficiency. Consistently, although they have never been functionally tested, heterozygous missense variants of *OSMR* or *IL31RA* have been associated with FPLCA (26, 27). An association of FPLCA with atopic dermatitis has also been reported (28). However, the role of IL-31 receptor biology in patients with AR OSMRβ deficiency is not straightforward. The IL-31 axis is strongly implicated in human pruritus and atopic skin inflammation, as illustrated by the efficacy of nemolizumab, an IL-31RA–blocking monoclonal antibody approved for the treatment of moderate-to-severe atopic dermatitis and prurigo nodularis (42, 43, 44, 45). This therapeutic effect suggests that excessive, rather than deficient, IL-31 signaling can promote atopic skin disease. As IL-31 responses were not assessed directly in the patients, the relative contributions of impaired OSM signaling, altered IL-31R-dependent signaling remain unresolved. Together, these studies identify AR OSMRβ deficiency as a new IEI and extend the genetic dissection of STAT3-dependent disease (46) by highlighting the contributions of the stromal and epithelial compartments to human atopy and barrier immunity. They also have immediate diagnostic implications: OSMRβ deficiency should now be considered in the genetic evaluation of patients presenting with severe atopic disease, hyper-IgE, eosinophilia, and HIES-like features.

## Data Availability

No new data were generated or analyzed in support of this study.
